# A qualitative study of the perceptions and experiences of healthcare providers caring for critically ill patients during the first wave of the COVID-19 pandemic: A PsyCOVID-ICU substudy

**DOI:** 10.1371/journal.pone.0274326

**Published:** 2022-09-09

**Authors:** Fiona Ecarnot, Sandrine Lombion, Aurélie Pourrez, Alexandra Laurent, Alicia Fournier, Florent Lheureux, Mélanie Loiseau, Jean-Philippe Rigaud, Christine Binquet, Nicolas Meunier-Beillard, Jean-Pierre Quenot

**Affiliations:** 1 Department of Cardiology, University Hospital, Besançon, and EA3920, University of Burgundy-Franche-Comté, Besançon, France; 2 EURL SLc, Chamesey, France; 3 Unité de Recherche UR3476, Mediation Research Center, University of Lorraine, Nancy, France; 4 Laboratoire de Psychologie: Dynamiques Relationnelles Et Processus Identitaires (PsyDREPI), Université de Bourgogne Franche-Comté, Dijon, France; 5 Department of Anaesthesiology and Critical Care Medicine, Dijon University Medical Centre, Dijon, France; 6 Laboratoire de Psychologie, Université de Bourgogne Franche-Comté, Besançon, France; 7 Service de Médecine Légale CHU Dijon, Cellule d’Urgence Médico-Psychologique de Bourgogne Franche-Comté, Dijon, France; 8 Service de Médecine Intensive-Réanimation, CH de Dieppe, France; 9 Espace de Réflexion Éthique de Normandie, Université de Caen, Caen, France; 10 Inserm CIC 1432, Module Épidémiologie Clinique (CIC-EC), CHU Dijon-Bourgogne, UFR des Sciences de Santé, Dijon, France; 11 Inserm CIC 1432, Clinical Epidemiology, University of Burgundy, Dijon, France; 12 DRCI, USMR, Francois Mitterrand University Hospital, Dijon, France; 13 Service de Médecine Intensive-Réanimation, CHU Dijon-Bourgogne, Dijon, France; 14 Equipe Lipness, Centre de Recherche INSERM UMR1231 et LabEx LipSTIC, Université de Bourgogne-Franche Comté, Dijon, France; 15 Espace de Réflexion Éthique Bourgogne Franche-Comté (EREBFC), Dijon, France; George Mason University, UNITED STATES

## Abstract

**Background:**

Intensive care unit (ICU) staff have faced unprecedented levels of stress, in the context of profound upheaval of their working environment due to the COVID-19 pandemic. We explored the perceptions of frontline ICU staff about the first wave of the COVID-19 pandemic, and how this experience impacted their personal and professional lives.

**Methods:**

In a qualitative study as part of the PsyCOVID-ICU project, we conducted semi-structured interviews with a random sample of nurses and nurses’ aides from 5 centres participating in the main PsyCOVID study. Interviews were recorded and fully transcribed, and analysed by thematic analysis.

**Results:**

A total of 18 interviews were performed from 13 August to 6 October 2020; 13 were nurses, and 5 were nurses’ aides. Thematic analysis revealed three major themes, namely: (1) Managing the home life; (2) Conditions in the workplace; and (3) the meaning of their profession.

**Conclusion:**

In this qualitative study investigating the experiences and perceptions of healthcare workers caring for critically ill patients during the first COVID-19 wave in France, the participants reported that the crisis had profound repercussions on both their personal and professional lives. The main factors affecting the participants were a fear of contamination, and the re-organisation of working conditions, against a background of a media “infodemic”.

## Introduction

The worldwide pandemic caused by the SARS-CoV-2 virus has put intensive care units (ICUs) around the world under unprecedented pressure, as they provide front-line care for the most severely ill individuals, notably those with acute respiratory distress requiring mechanical ventilation. The clinical and technical expertise of ICU staff across the globe has been widely recognized during the present crisis. However, many hospitals were (and remain) submerged by the various waves of COVID-19, and in many places, there were shortages of beds, ventilators and medications in ICUs, but also a shortfall of staff to meet the needs of these severely ill patients receiving life-support therapies. In this context, ICU staff have been faced with exceptionally high levels of stress, while at the same time, their working environment has undergone profound upheaval. Additionally, healthcare workers experienced exposure to constant media coverage (so-called “infodemic”). They had doubts and uncertainties surrounding the right treatments to give patients. They were at risk of contamination at work and at home, and experienced serial lockdowns and social restrictions. The repercussions of these factors on all healthcare professionals are becoming evident, and particularly the impact on healthcare workers in the ICU. Azoulay et al. reported that 50.4% of healthcare workers in the ICU had symptoms of anxiety, 30.4% had symptoms of depression, while 32% had peritraumatic dissociation [1]. In a recent review of the literature that included 13 studies totalling 33,062 (mostly Chinese) healthcare workers, the authors found a pooled prevalence of around 23% for both anxiety and depression across studies [2].

In the PsyCovid-ICU study, our group previously showed during the first pandemic wave in 2020 that healthcare professionals working in zones where the pandemic intensity was highest were at greater risk of mental health issues, with higher levels of perceived stress [3]. Risk factors for this psychological distress identified in the study, regardless of the pandemic intensity, were female sex, having a relative at risk of COVID-19, and working in geographical zones where the pandemic was of greatest intensity. Furthermore, certain categories of ICu professionals, such as those closest to the patient (nurses, medical interns, nurses’ aides), but also staff who did not usually work permanently in the ICU (i.e. reinforcements deployed to the ICU during the crisis) were also found to have higher levels of stress.

To complement and elucidate the quantitative findings of the PsyCOVID-ICU study, we sought to explore the perceptions of frontline ICU staff about the first wave of the COVID-19 pandemic, and how this experience impacted on their personal and professional lives, in a qualitative study using semi-structured interviews.

## Methods

This qualitative study was performed as part of the larger PsyCOVID-ICU study, whose methods and results have previously been reported [3]. Briefly, the PsyCOVID-ICU study was a large, prospective study conducted in 77 hospitals across France from 22 April 2020 to 13 May 2020. The population of the main PsyCOVID-ICU study comprised ICU frontline healthcare workers directly involved in the diagnosis, treatment, and care of patients with COVID-19 (i.e., physicians, residents, nurses, nurses’ aides, medical students and nursing managers) who consented to participate. An online questionnaire was sent using the Limesurvey platform, and participants first provided demographic data, then completed the 12-item General Health Questionnaire (GHQ-12) for the evaluation of the primary endpoint, which was mental health. Participants also completed the Perceived Stressors in Intensive Care Units (PS-ICU) scale to measure sources of stress in ICU during the COVID-19 crisis. A total of 2,643 healthcare professionals were included, of whom 72.6% were females, and 53.2% were nurses.

The PsyCOVID-ICU study received approval for all participating centers from the Ethics Committee of the French Intensive Care Society (N°20–33), for both the quantitative and qualitative parts. Consent to participate in the main PsyCOVID study was implied by the fact that all participants voluntarily connected to the study website and completed the forms. Participants also agreed to participation at the beginning of the survey before proceeding with completion of the forms, and provided consent to be contacted for a qualitative interview to explore their responses in greater detail.

For the present qualitative study, a random sample of 5 hospitals (Strasbourg, Dijon, Vesoul, Metz-Thionville, Argenteuil) were drawn from among all the participating centres. Then, among these centres, a random sample of 10 participants (nurses and nurses’ aides) was drawn from among the participants who had consented to be contacted for an interview. It was estimated that the response rate from a sample 50 (10 participants to be contacted from each of 5 centres) would be sufficient to achieve data saturation. Participants from each centre were contacted by a researcher from the Psy-COVID-Qualitative team (NMB, male; FE, SP, SL, female) to organize an individual semi-structured interview at a time convenient for the interviewee. We developed an interview guide focused on three main topics, namely: (1) experience of the pandemic crisis and its effects on their personal life (e.g. domestic life, social and family relations); (2) effect of the pandemic on their professional life (e.g. work organisation, resource management, relationship with the hierarchy/ administration/ colleagues); (3) societal aspects of the pandemic (e.g. role of the media and/or experts, public opinion). The interview guide was developed by our group based on a review of available literature, plus a focus group with a sociologist, qualitative researchers, and ICU physicians and nurses.

Due to the ongoing pandemic during the study period, all interviews were performed by telephone. Interviews were performed until data saturation was reached. Interviews were recorded and fully transcribed (in French) for later analysis. Data were encoded to guarantee the anonymity of the participants.

The transcripts of all interviews were analysed using thematic analysis, as previously described [4, 5]. The first level of analysis was first performed independently by each researcher (FE, SL, AP, NMB) on their own interviews, then meetings were held to harmonize the categories and themes to be retained. Results were discussed until consensus on interpretation was reached. Translation was performed after the results were finalized. Participants were informed that citations from their discourse may be used (translated into English) to illustrate the results of the study in a scientific publication, and all participants consented to this.

## Results

Data saturation was reached and verified after a total of 18 interviews, which were performed from 13 August to 6 October 2020; 13 were nurses, and 5 were nurses’ aides. The characteristics of the study population are shown in Table 1.

**Table 1:** Characteristics of the study population (N = 18).

Participant	Age group	Gender	Job	Years work experience	Marital Status	Children?
P1	35–49 years	Female	RN	7 years	Single	No
P2	20–34 years	Female	RN	9 years	Living maritally	No
P3	20–34 years	Female	RN	9 months	Single	No
P4	35–49 years	Female	NA	6 years	Married	Yes
P5	20–34 years	Male	RN	18 months	Living maritally	No
P6	20–34 years	Male	RN	18 months	Single	No
P7	35–49 years	Male	RN	Unknown	Living maritally	Yes
P8	50–65 years	Female	NA	30 years	Married	No
P9	35–49 years	Male	RN	17 years	Married	Yes
P10	35–49 years	Female	RN	16 years	Married	Yes
P11	20–34 years	Female	RN	11 years	Living maritally	Yes
P12	35–49 years	Female	NA	2 years	Married	Yes
P13	20–34 years	Female	NA	12 years	Married	No
P14	20–34 years	Female	RN	15 years	Married	Yes
P15	35–49 years	Female	RN	11 years	Married	Yes
P16	20–34 years	Female	RN	4 years	Single	No
P17	35–49 years	Female	AS	Unknown	Single	Yes
P18	20–34 years	Female	RN	1 years	Single	No

RN, Registered nurse; NA, nurses’ aide.

Thematic analysis of the interviews revealed three major themes.

**Fig 1:**
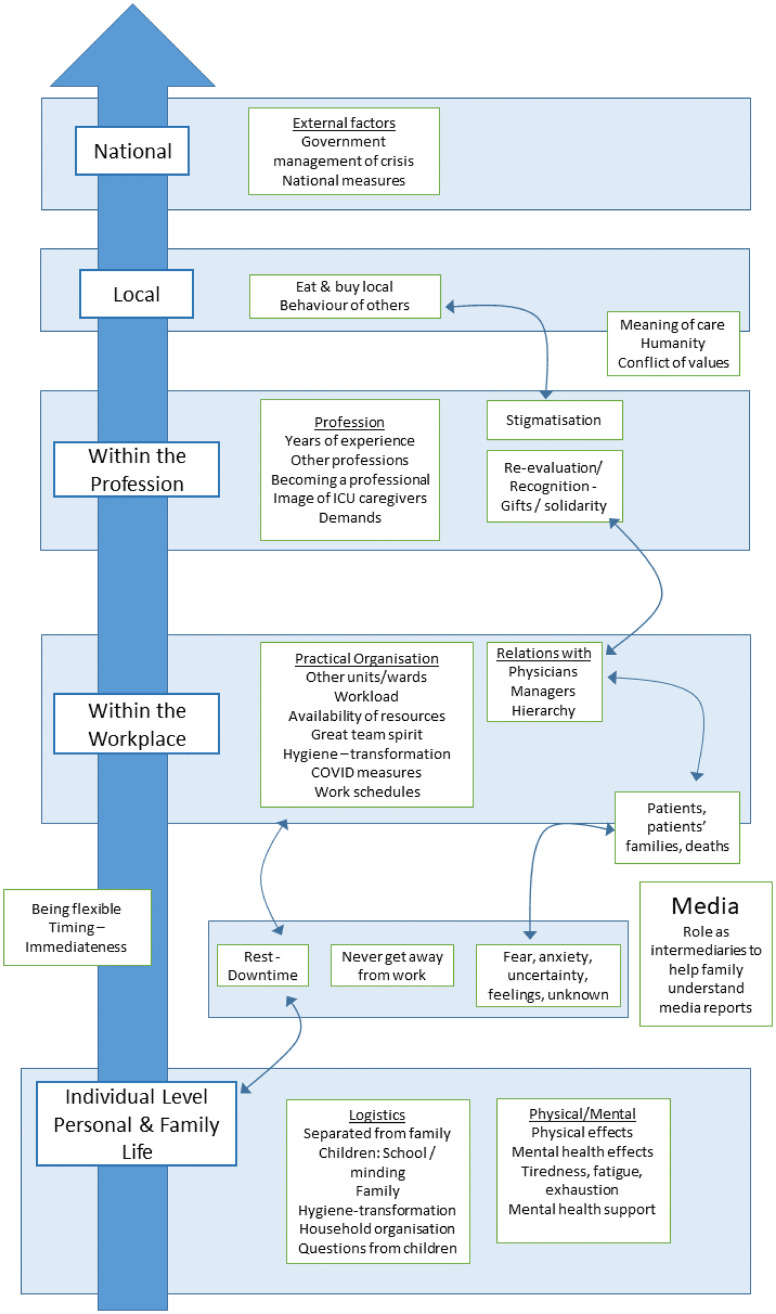
Conceptual framework describing the main themes and the relations between them.

Illustrative quotes are given in italics for each point. The conceptual framework describing the themes and their interrelations is illustrated in Fig 1.

### Managing the home life

Firstly, the main theme raised by all participants was the management of their personal/home life. This included several logistic challenges in the homeplace such as juggling with home-schooling for their children while still working themselves. In terms of support from their families, some participants had no support as they were separated from their families, either by choice or by necessity, due to the pandemic, whereas others had strong support from a spouse (doing shopping, housework etc), which enabled them to continue working.

*"We shared the household tasks*. *My husband*, *when he was working from home*, *he’d do the homework with our boy*, *while I mostly looked after the two girls*. *I did the cleaning or we’d share it*, *whoever was available would do a bit of housework*. *Same for the meals*.*”*

*“I didn’t get much rest*. *I live alone*, *not too far from my parents*, *but they’re getting old*, *and my father is sick*, *and since apparently there’s no way to know if you’re a healthy carrier [of COVID-19]*, *well I knew that if he got COVID*, *he’d die*, *so I chose not to have any contact with my family*. *So basically*, *I was on my own for 2 months*. *Sure*, *they understand*, *and we stayed in touch by phone*. *But they had a better time of it than I had during the lockdown*, *because they’re retired*, *because they’re at home*, *and they don’t have to work”*.

*“My daughters helped out*. *They did some cleaning and a bit of cooking*. *No*, *really*, *they were great about that”*

All participants underlined the major attention they paid to hygiene, notably with a complete change of clothes before entering the home, showering, etc.

*“I would have a shower in work*, *and change my clothes*. *Then I put them in a sack*, *went home and put them directly into the washing machine*, *and the bag into the furnace*, *and then I would have a shower again”*.

Finally, many of the participants noted that their children asked a lot of questions, which they were sometimes unable to answer.

*“My big lad*, *he’s 11*, *he clearly understood everything*. *The smaller two*, *not so much*. *The middle boy would ask questions*, *we tried to answer as best we could so he would understand”*

In terms of the personal life, the participants also underlined the physical and mental effects of the pandemic. This was manifested by persistent fatigue and other physical symptoms, and in some cases, the participants also felt a need for psychological support.

*“My husband didn’t take it well*, *he was upset*, *had a pain in his stomach*, *couldn’t eat*, *couldn’t sleep”*

*“We all suffered mentally and physically*. *[…] COVID made me lose …*. *well*, *at times*, *lose hope*. *[…] Several of our colleagues are in burn-out”*.

### Conditions in the workplace

The second major theme concerned the working conditions during the pandemic. The primary point underlined by all participants in this regard was the increased workload, the rapid influx of patients, and the availability (or not) of the resources needed to deal with the crisis.

*“the first wave started in [two cities located further south]*, *and when they were full*, *we started taking their patients*. *But then*, *the wave hit us*, *and we were already full*, *and there were all these patients arriving…*‥*”*

*“The most particular thing was that all my patients had the same disease*. *That never happens*! *All of them had the same disease*, *and they were all arriving at the same time and we had to re-organise to be able to manage*. *It was all the same thing*, *and too many of them all at once”*.

*“Well we just had to get organized*. *The operating theatres closed and the recovery rooms were used as ICUs*. *Lots of other departments re-opened as COVID units*. *The reorganisation was quite impressive”*

There was an increased burden of logistics in terms of hygiene and personal protection measures. Overall, while there was no lack of protective material (gowns, gloves, masks) in the intensive care units of our study participants, there was rationing. Our study participants recognized that in this regard, they were better equipped than some of their colleagues in other hospital departments.

*“We had equipment*, *and we got gifts*. *There were dressmakers who made gowns for us because we were running out*. *We always had masks although it’s true that at the beginning*, *they were rationed*. *We were careful not to waste but we had enough*.*”*

*“I fainted once*, *hypoglycemia*. *The equipment was unbearable to wear but at least we were lucky enough to have [personal protective equipment]*, *it’d be ungrateful of us to complain*, *compared to others who had nothing”*

As regards the practical aspects of the work schedules, many participants pointed out that extra staff were brought in for the pandemic, which actually made their working conditions somewhat easier, as the nurse-to-patient ratio was reduced. In addition, since visits were forbidden, the time spent meeting and discussing with patients’ families was also redirected to other tasks.

*“The doctors would call the families*, *depending on the patient’s situation*. *So during the whole time*, *we had practically no contact with families*. *That was a huge relief*, *not to have to deal with the families on top of everything else”*.

*“I know that what I’m going to say isn’t nice*, *but it’s easier to do your job [when the families are not there]*, *you’re not being interrupted all the time to run and open the door*, *and answer their questions right that very minute… you know what it’s like”*

However, having to train the additional staff brought in to cover the increasing workload was seen as a burden by some participants. Several participants also underlined that while the extra help was welcome during the peak of the first wave, the extra staff were rapidly re-deployed as soon as the lockdown restrictions were lifted at national level. The participants would have welcomed a prolongation of these reinforcements in their everyday work.

*“The fact that there were more of us*, *yeah*, *that was really a bonus*, *it was great*. *We were able to work safely*, *and that was really good*. *But you know… quotas are what they are*! *As soon as things calmed down*, *as soon as the COVID slowed down*, *that was the first thing they took away”*

*“We called in nurses who already had some ICU experience*, *but there were one or two who had no experience…*. *It was hard for them because*, *well*, *we had a huge amount of work and we couldn’t train them as well as we should have*, *and so I think that overall*, *they had a harder time of it than we did”*.

Overall, all the participants were unanimous in underlining the strong solidarity and cohesion in their work team, whereby everyone really pulled together to get through the crisis. Several of them also highlighted that they felt a sort of adrenaline rush and excitement that they had not felt for a long time, or that gave them the motivation to keep going even when they felt exhausted.

*“Most of my colleagues*, *they’re like a second family for me*, *and I think I can speak for everyone when I say that”*

*“It was really hard*, *physically… mentally…*. *But I really felt that I was fighting for something”*

*“Not many of them survived*, *but at least it brought back to me why…*. *Why I work in the ICU*, *why I chose the ICU”*.

The question of interactions with the hierarchy in the workplace was also to the forefront in the participants’ discourse about their experience of the pandemic in the workplace. Generally, the nursing managers and immediate superiors were present all of the time and strongly engaged in helping the staff as much as possible. Interactions with more superior levels of the hierarchy (e.g. hospital administration) were perceived to be more difficult, and some frictions between nurses and physicians were mentioned. However, overall, the prevailing sentiment was one of solidarity and cohesion in the face of an unprecedented crisis.

Moreover, in the workplace, the relations with the patients and their families were an important point for all the participants. They reported the difficulty of announcing bad news to families by telephone, of not being able to accompany grieving families in the usual way. They felt that refusing to give families access to their deceased loved-ones was a failure to act in a humane manner.

*“Usually*, *visits are allowed 24/7 in our unit… Then overnight*, *visits were totally forbidden*, *we had to put everybody out at once*. *It was very stressful for them*, *and for us*, *from one day to the next families were no longer allowed to come in and had to just wait by their phone*, *in lockdown*, *waiting for the physician to call with news of their loved one*. *I can just imagine all those families out there*, *with nothing to do only wait*, *wait*, *wait beside their phone”*

*“The deaths were complicated*, *especially in the early days*, *because you’d think to yourself*, *well*, *I’m the last face this person will ever see*. *And we’re not even their family”*

They also reported very special relations with the patient due to the intensity of the care process, the isolation of the patients and the unusually long duration of the ICU stay. Indeed, many of them reported having maintained links with surviving patients, who came back after their ICU stay to thank the staff and give news of their outcome.

Mediating between both the home life and the workplace, there was an emotional context that was fed by both home and work experiences, but which simultaneously affected both the homelife and the work life. There was a permanent fear of contamination, notably of contaminating their families from an infection caught in the workplace, or of falling ill themselves. This was exacerbated by the prevailing uncertainty surrounding the disease, and the unprecedented nature of the situation. This constant fear made it difficult for the study participants to “switch off”, even when they were not on duty. In addition, the lockdown conditions meant that they could not engage in their usual leisure activities, so “down-time”, relaxing and leisure became difficult to achieve.

*“We were getting conflicting information…[…] the instructions were changing almost every hour*. *It makes you very nervous and very anxious”*

*“I need…*. *It reassures me to know what to expect*. *But with this*, *you just had to dive into the unknown and say to yourself*, *OK*, *I don’t know if I’m going to be contaminated*, *I don’t know if I’ll be able to leave*, *I don’t know what tomorrow will bring*, *I don’t know if we’ll ever be free again*, *I don’t know if the day will come when I can go home from without having a pain in my head from breathing through a mask for 8 hours*. *Personally*, *it really disturbed me”*

*“The gym where I used to go was closed*. *Everything was closed*. *I slept a lot*.*”*

*“Rest… that was kind of complicated*. *Let’s just say we didn’t get much downtime”*

Finally, the media also played a major role in perpetuating the climate of fear and uncertainty. Many of the participants were called on by their families to answer questions about things they heard on the television or radio, meaning that even in their free time, they were still caught up in the pandemic, as if they were at work.

*“They didn’t see the reality on the ground*. *They only had the information they got from journalists and the media and the internet*, *and there was so much information that*, *…*. *Well… it penalized us because we had families insulting us on the phone because we didn’t do this or that*, *or they heard some doctor say this or that…*‥ *you see*? *There was too much information and since people were stuck at home in lockdown*, *they had plenty of time for it*!*”*

### The meaning of the profession

The combined experience of working through the pandemic and managing their homelife against the emotional background of the lockdown, prompted the study participants to reflect at a more abstract level about the meaning of their profession, and this was the third theme to emerge from the analysis. In particular, it led them to reflect on what it means to be a healthcare worker in critical care, particularly because the media portrayed a certain image with which some caregivers did not identify. In this regard, the term “hero”, often advanced by the media, did not sit well with our study participants, who felt that far from being heroes, they were simply doing their job.

*“Heroic*? *Well*, *that’s going a bit too far”*

*“It’s our job*. *We do it with our heart and soul*. *We didn’t feel… well*, *the medals…*. *Why are there medals now*? *The medal is all year long*. *All year long*, *we have to work on a shoestring*, *but then for COVID*, *it was open-bar for everything”*

Some participants mentioned that the pandemic revived in them the excitement and motivation of their early career, while others mentioned that they might consider a change of discipline after the pandemic, having “had their fill” of critical care. Indeed, the pandemic led some participants to reflect on the values they hold, as some reported that what they experienced in the workplace during the crisis went against the values they personally held dear. The experience of the crisis prompted some participants to reflect on what humanity means to them.

*“It was awful to see all these people dying in absolutely dreadful conditions*, *people who wouldn’t have died in normal times*, *people with no health problems…*.*There you go*, *it was… we have a lot of deaths in the ICU ordinarily*, *but not in conditions like that*, *not dying one after the other*, *not having to triage*, *to choose*. *I’m going to speak crudely*, *but it was like as if we had to choose who lives and who dies*. *And that was really terrible”*

Since the pandemic focused the media spotlight on the profession of critical care health workers, many participants felt that the profession as a whole should capitalize on this positive media attention to achieve a new level of recognition for their profession, for example by raising the status of critical care nurses to the same degree of professional qualification as that accorded to other nursing specialties.

*“In the heat of the action*, *they put you on a pedestal*, *but then afterwards*, *they forget about you”*

*“I was happy that people could finally discover our specialty*, *what it means to work in the ICU”*

*“We need to be recognized*, *recognized and appreciated*, *for our competences and given the appropriate degree of recognition with the corresponding salary”*

*“This Federation [note*: *federation of ICU nurses created during the pandemic] can finally take action*, *and do some lobbying to get recognition for our specialty”*

All the participants were amazed by the outpouring of generosity from the public, and they reported, for example having received enormous amounts of free meals, gifts, and services from local businesses, shops, restaurants, schools and residents.

Finally, two minor themes also emerged from the interviews in this study. Firstly, there was a raised awareness of the importance of local businesses, eating locally produced produce, and behavioural interactions with people in the local environment. Secondly, most participants noted that their experienced of the crisis always played out against the national background, namely the management of the pandemic by the government, the measures implemented at national level, etc.

## Discussion

This qualitative study investigating the sources and repercussions of the psychological distress suffered by healthcare workers during the COVID-19 pandemic, provides novel insights into the perceptions and experiences of ICU staff, and how this affected them in their daily personal and professional lives. There is paucity of data on this topic, yet the health of these professionals seems to be in jeopardy, with a level of psychological stress that has been cumulating over the course of pandemic, and reports of a fear of dying and an elevated risk of suicide [2].

Reports to date about the mental health in healthcare professionals have mainly relied on quantitative data, with wide heterogeneity across studies due to the large number of different scales and tools used. As a result, comparisons are difficult, and it is difficult to draw conclusions about the individual effects of various factors in generating anxiety, depression or post-traumatic stress disorder [1–3, 6]. This is an important issue, because although quantitative scales and validated instruments are necessary to identify and quantify the severity of psychological distress in specific populations and environments, they do not take account of each individual’s personal context, thereby also precluding individualized follow-up, as has been proposed by some authors in the framework of mental health service reforms [7, 8]. Our study therefore aimed to explore the individual consequences of the psychological distress perceived by healthcare workers during the pandemic in France, exploring both their personal and professional lives, and to put these consequences in perspective with literature data and avenues for instigation of mental health support services.

Our findings underline that the personal and professional domains are intricately linked, and difficult to dissociate. The respondents reported their fear of carrying contamination from the workplace to the home and family. This fear was compounded by the national lockdown, which forced families to reduce contacts outside the home, with children on home-schooling. The restrictions obliged many healthcare workers to improvise solutions to meet the family’s needs within the limits of the resources available to them. Some, but not all were able to count on the presence of a spouse or other family member to help out in the home. Many healthcare workers reported having instituted their own “decontamination zone” in the home, where they would shower and change on arrival after work to avoid contaminating their entourage. The role of the media, and the COVID-19 “infodemic”, stands out in our results as a new phenomenon faced by healthcare workers on their days off, making it difficult for them to disconnect from their work, thereby compounding their sense of fatigue, stress and anxiety, and hampering their ability to rest and “refuel”.

Many of these fears have been shared by healthcare workers worldwide since the beginning of the crisis, notably with the first reports from China soon after the onset of the pandemic [9, 10]. These studies identified the feeling of uncertainty and insecurity as being problematic, with healthcare workers afraid of being contaminated in the workplace, and also afraid that they would in turn contaminate their entourage [11]. This fear is even greater for healthcare workers who have vulnerable individuals in their entourage, such as family members with a chronic disease, and this may translate into symptoms such as insomnia or anxiety [12].

The lockdowns imposed in many countries to limit the spread of the pandemic may have led to feelings of loneliness and isolation, which can in turn generate anxiety and/or depression [13]. The COVID-19 infodemic, with its abundance of information on all the media outlets and social networks, may have engendered fear, or even panic, inciting forms of suspicions, discrimination and stigmatization due to the risk of contamination [14]. All these circumstances have had direct repercussions on the personal lives of healthcare workers [15–17].

Conversely, some positives effects have also been reported, including the benefits of social support, correlated with self-efficacy (the individual’s belief in their ability to accomplish a given task), and sleep quality, and a decrease in anxiety and stress [18]. The scientific advisory board advising the French government through this crisis was quick to recommend the implementation of psychological support units in hospitals nationwide in collaboration with the existing network of medico-psychological emergency units, usually mobilized to help victims of large-scale events such as terrorist attacks or natural disasters [19–21]. Other *ad hoc* solutions also emerged, notably the recognition that mental health services should be provided for the family and friends of people affected by the COVID-19 crisis [22]; the online delivery of psychotherapy or psycho-educational services, or the use of internet and social media to share strategies for coping with psychological stress [7, 8, 23]. Recent research has indicated the Eye Movement Desensitization and Reprocessing (EMDR) and cognitive behavioural therapy may be efficacious for reducing the symptoms of post-traumatic stress disorder [24, 25]. However, it is not unreasonable to consider that the services provided are likely insufficient, in the face of such a monumental pandemic. A more holistic approach to mental health at work is needed, with institutional support for resilience, and a hierarchy that takes account of emotional distress, as well as organisational and relational difficulties among the caregiving staff [26].

Within the workplace, the reorganisation of care delivery (in terms of structure and human resources) was sometimes viewed in a positive light, with extra staff brought in to help, and more relaxed rules (greater presence of management, additional supplies etc). Conversely, others perceived these changes in a more negative light, due to the increased workload from having to train support staff, or due to the absence of the patients’ families during the lockdown. Lost opportunities for care due to a shortage of ICU beds were rapidly identified by many healthcare workers as possible major issue for the pandemic, leading the French Intensive Care Society to issue recommendations about admission criteria to the ICU for the pandemic context as well as recommendations for allowing family visits in the ICU (www.srlf.org) [27].

Healthcare workers received a good degree of recognition from the public during the first wave of COVID-19 infections, and in France, there was applause in public places every evening at 8pm. It has been reported that social support can help build resilience and promote recovery after stressful encounters [28, 29]. As underlined by Chen et al., concrete measures to provide support to healthcare workers (e.g. specific break rooms, and the possibility to have some “down-time”) are the most appropriate for healthcare workers during a crisis [19]. The development of preventive strategies is also necessary to support healthcare workers continuing to care for severely ill patients, thereby protecting a resource that is increasingly precious in this ongoing crisis [30, 31].

All the participants in this study underlined the strong solidarity and team spirit they experienced, with a renewed sense of purpose and an “adrenaline rush” that motivated them to keep going when things were difficult. Some healthcare workers use this as a coping strategy in the face of stress and demanding situations. In the PsyCOVID-ICU study, the use of positive re-evaluation as a coping strategy was shown to have a positive effect on mental health. Indeed, experiencing positive emotions promotes resilience, and reinforces certain cognitive functions, such as attention, creativity and flexibility [3, 19, 26, 28].

This study has some limitations. First, the questions asked during the interview may not have covered all the aspects of the crisis, or the full spectrum of repercussions that the pandemic may have had on all areas of their personal and professional lives. Secondly, only healthcare workers who had participated in the quantitative PsyCOVID-ICU study and who agreed to participate in the qualitative study were interviewed. Third, due to the lockdown and restrictions in place, the interviews were performed by telephone, which may have influenced the respondents’ responses as compared to face-to-face interviews. Lastly, only French healthcare workers were included, and the experiences of front-line workers in other countries would undoubtedly be different.

## Conclusion

The healthcare workers in this qualitative study expressed their personal concerns and perceptions of the COVID-19 crisis, as experienced during the first wave of the pandemic. There were far-reaching effects on both the personal and professional lives, linked to a fear of contamination, and to the re-organisation of working conditions, against a background of the media “infodemic”. Positive effects, such as renewed motivation for their work, closer team spirit, and greater resilience, were also mentioned. Impact studies, both in the prevention and treatment of mental health issues among healthcare workers could be useful to avoid long-term psychological distress due the ongoing pandemic in this category of professionals.
